# The severity of respiratory syncytial virus infection in children during the SARS-CoV-2/COVID-19 pandemic: A nationwide study of 11,915 cases in Germany

**DOI:** 10.1007/s15010-024-02385-9

**Published:** 2024-09-10

**Authors:** Sarah Maslowski, Sven Hohenstein, Andreas Bollmann, Christian Karagiannidis, Cihan Papan, Serge C. Thal, Stefan Wirth, Tobias Tenenbaum, Malik Aydin

**Affiliations:** 1 Department of Anesthesiology, Center of Clinical and Translational Research (CCTR), Helios University Hospital Wuppertal, Witten/Herdecke University, Wuppertal, Germany; 2 Helios Health Institute, Leipzig, Germany; 3 Department of Pneumology and Critical Care Medicine, ARDS and ECMO Center, Kliniken der Stadt Köln gGmbH, Witten/Herdecke University Hospital, Cologne, Germany; 4 Institute for Hygiene and Public Health, University Hospital Bonn, Bonn, Germany; 5 Center for Child and Adolescent Medicine, Center for Clinical and Translational Research (CCTR), Helios University Hospital Wuppertal, Witten/Herdecke University, Wuppertal, Germany; 6 Clinic for Child and Adolescent Medicine, Sana Klinikum Lichtenberg, Academic Teaching Hospital of Charité-Universitätsmedizin, Berlin, Germany; 7 Laboratory of Experimental Pediatric Pneumology and Allergology, Center for Biomedical Education and Research, School of Life Sciences (ZBAF), Faculty of Health, Witten/Herdecke University, Witten, Germany; 8 Virology and Microbiology, Center for Biomedical Education and Research, School of Life Sciences (ZBAF), Faculty of Health, Witten/Herdecke University, Witten, Germany; 9 Institute of Medical Laboratory Diagnostics, Center for Clinical and Translational Research (CCTR), Helios University Hospital Wuppertal, Witten/Herdecke University, Wuppertal, Germany

**Keywords:** RSV, Respiratory Syncytial Virus, Infection, Virus, Children, SARS-CoV-2, COVID-19

## Abstract

**Purpose:**

Respiratory syncytial virus (RSV) infection is a major cause of childhood hospitalization. The COVID-19 pandemic has disrupted the usual seasonal pattern of RSV, resulting in high activity during the off-season. This study aims to evaluate the effects of the pandemic on the severity of RSV infections.

**Methods:**

Data from 11,915 children hospitalized due to RSV infection between 2016 and 2022 were analyzed. The hospitalized patients were categorized into two groups, from January 2016 to February 2020 (PreCoV19 group) and from March 2020 to December 2022 (CoV19 group). The hospitalization duration, intensive care unit (ICU) admissions, length of stay at ICU, mechanical ventilation requirement and duration, Elixhauser comorbidity index scores, and in-hospital mortality were analyzed.

**Results:**

Children in the PreCoV19 group had a mean age of 0.4 ± 0.7, whereas those in the CoV19 group had a mean age of 0.6 ± 1.0 years. Children during the pandemic had significantly shorter hospital stays (4.3 ± 2.6 days) compared to children of the pre-pandemic period (4.9 ± 3.3 days). Although ICU admission rates did not change, the duration of ICU stays decreased in the CoV19 group. Moreover, the in-hospital mortality did not differ between the groups. A multivariable analysis showed that younger age, regardless of the pandemic period, was associated with prolonged hospital stays, higher ICU admission rates, and an increased requirement for mechanical ventilation.

**Conclusion:**

Our findings highlight significant changes of the clinical characteristics of RSV infections during the pandemic, with implications for clinical management and public health strategies.

**Supplementary Information:**

The online version contains supplementary material available at 10.1007/s15010-024-02385-9.

## Introduction

Respiratory syncytial virus (RSV) is the most common viral pathogen causing acute lower respiratory tract infection in children, significantly contributing to increased morbidity and mortality [1]. In particular within the first five years of life, RSV infection is one of the leading causes of hospitalization, resulting in 3.6 million hospitalizations globally in this age group [2]. Characteristically, RSV infections follow a recurrent seasonal pattern in the northern hemisphere, starting in October/November, reaching a peak incidence in January, and concluding by March/April [3]. However, RSV epidemiology and seasonality were interestingly disrupted by the onset of the severe acute respiratory syndrome coronavirus 2 (SARS-CoV-2), which caused the coronavirus disease 2019 (COVID-19) pandemic in March 2020 [4].

The implementation of non-pharmaceutical interventions, including ‘lockdowns’, social isolation, increased viral testing, mask-wearing, and disinfection routines, resulted not only in a reduction of COVID-19 infection incidence, but also in a significant reduction of other seasonal viruses e.g., influenza virus, rhinovirus, or RSV [5]. A particular decrease of 98% in RSV infections was also observed worldwide [6]. In Europe and the USA, studies demonstrated an abrupt and early end of the RSV season after the pandemic has been declared by the World Health Organization (WHO) [7–10]. In the southern hemisphere, the implementation of preventive measures coincided with the onset of the winter, resulting in a complete absence of the RSV season [11]. In Germany, a laboratory-based national surveillance reported a decline in almost all respiratory-transmitted infectious diseases shortly after the implementation of non-pharmaceutical interventions in March 2020 [12]. The Robert Koch Institute, which is the Germany’s official public health institution for disease control and prevention, similarly reported a decline in RSV infections starting in calendar week 10 of the year 2020, and a complete absence of RSV infections until the calendar week 9 of 2021 [13].

As restrictions were reduced, there was an increase in RSV activity which was observed globally [14]. For the first time, an RSV season presented outside its usual seasonal pattern with significantly higher viral activities in both hemispheres [15, 16]. In German children’s hospital, a Clinician-Led Reporting System established in October 2021 documented a significant increase in RSV infections during the late fall and early winter of the 2021/2022 season, reaching the highest numbers since 2009 [17]. The RSV season of 2022/2023 also showed an unexpected increase in cases by early November, exceeding the numbers of the previous year [18].

Beyond the impact on the viral prevalence and seasonality of RSV infections, studies from Australia [19], the USA [20], France [21], and Denmark [22] demonstrated pandemic-associated alterations in disease severity and age distribution. Few studies reported higher proportions of severe infections among younger children, with increased hospital admission rates of pediatric ICUs and longer hospital stays compared to the pre-pandemic [7, 18, 23]. Other studies, however, did not show significant changes in this regard, indicating inconsistent findings [24, 25].

Given the substantial burden of RSV infections in childhood, it is thus important to study whether the pandemic and its associated sociopolitical measures influenced not only epidemiologic characteristics but also the severity of RSV infections in susceptible patient groups such as children. This has significant implications for healthcare systems in response to the pandemic.

To evaluate the impact of the COVID-19 pandemic and its non-pharmaceutical measures on the severity of RSV infections in children, a nationwide, multi-center study was performed using data from a German hospital network.

## Materials and methods

### Study site

**Fig. 1:**
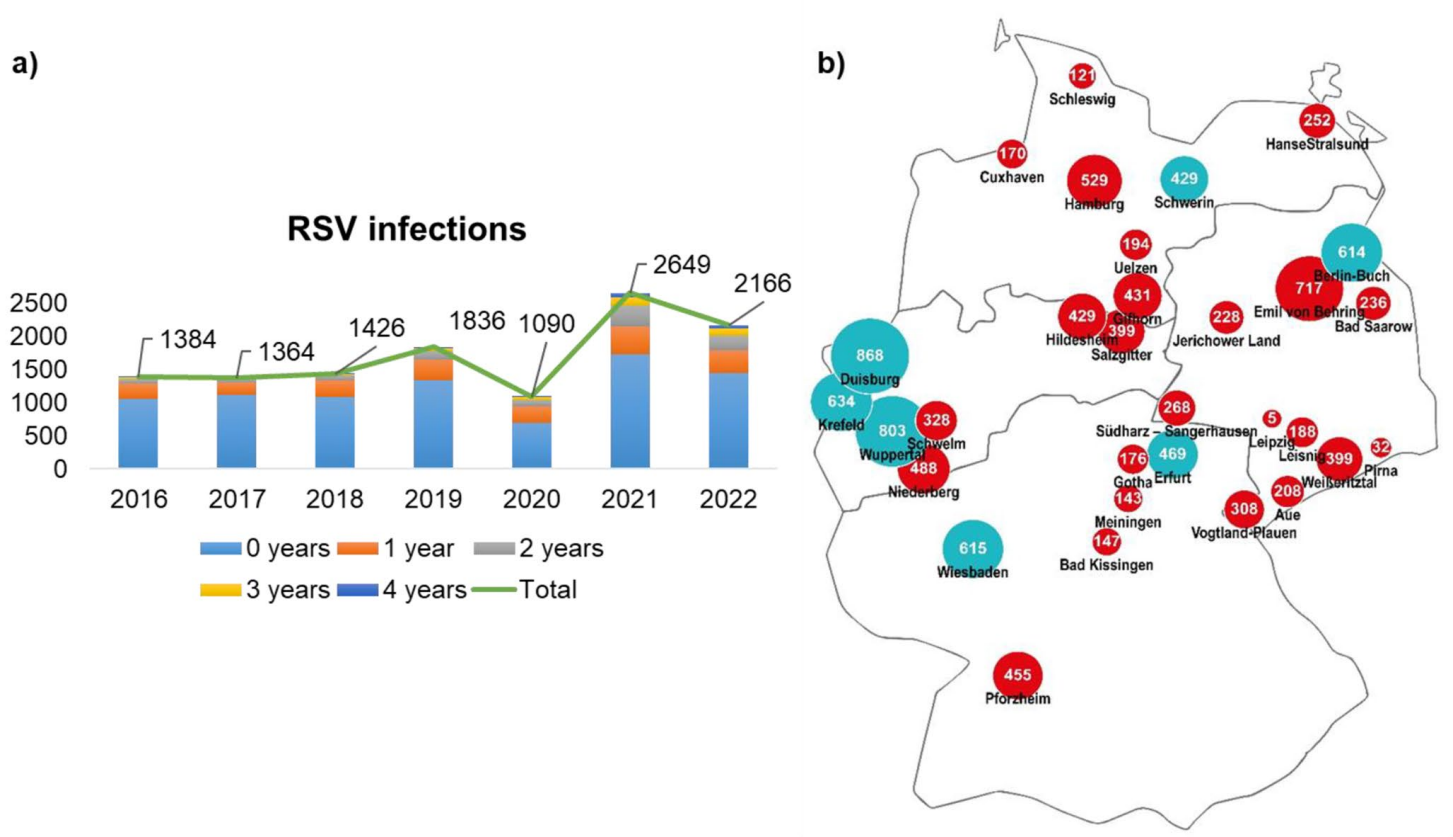
Descriptive presentation of pediatric cases with RSV infections between 2016 and 2022. (**a**) Timeline of recruitment per year, stratified by age group. (**b**) Map of the German hospital network. Blue dot: Tertiary care hospitals; Red dot: Secondary care hospitals; Dot size represents the number of included cases

A total number of 11,915 cases were analyzed from 31 German children’s hospitals, belonging to the Helios hospital network representing approximately 10% of the 305 hospitals with pediatric departments out of the 1,893 total hospitals in Germany [26], where these hospitals provide three levels of care (Fig. 1b).

Children under the age of five receiving full inpatient treatment for one of the following primary diagnoses and having the following ICD-10 diagnosis were included: i) acute bronchiolitis due to RSV infection (J21.0), acute bronchitis due to RSV (J20.5), and iii) RSV pneumonia (J12.1). The ICD-10 is a medical classification list developed by the WHO and represents the 10th revision of the International Statistical Classification of Diseases and Related Health Problems.

Hospital admissions from January 2016 to February 2020 were classified as pre-pandemic and were defined as the PreCoV19 cohort in this article. The admissions from March 2020 to December 2022 were categorized as the pandemic cohort, which correlates to the term CoV19, representing the beginning of the pandemic as also declared by the WHO (Fig. 1a).

An ethics approval was obtained from the ethics committee of the Witten/Herdecke University, Germany (S-18/2022). To maintain the flow of the text, the term SARS-CoV-2/COVID-19 pandemic will be replaced by ‘COVID-19 pandemic’ throughout the manuscript.

### Indicators of clinical severity

To evaluate the clinical severity of RSV infections in children, we analyzed the treatment duration and intensity. We documented the duration of hospitalization (number of nights spent in the hospital), admissions to the ICU, operationalized as procedure codes: 8-980 (complex intensive care), 8-98d (complex pediatric care), 8-98f (high-scoring complex intensive care), and the length of stay in ICU in days. We included the requirement for and duration of mechanical ventilation in continuous positive airway pressure (CPAP), high-flow oxygen, or invasive ventilation in hours, classified as procedure codes 8-70x (access during mechanical ventilation and using airway clearance techniques), 8-71x (mechanical ventilation and respiratory assistance through face mask or tube and weaning from mechanical ventilation), and in-hospital mortality. Moreover, we categorized the duration of mechanical ventilation into several tertiles: a) low (1 to 68 h), b) intermediate (69 to 123 h), and c) high (124 to 792 h). Variance tests were conducted to compare the variances between the PreCoV19 and CoV19 groups. To enhance the understanding of group characteristics, we also used the Elixhauser Comorbidity Index (ECI) to evaluate 30 comorbidities, which are relevant for pediatric and adult patients, providing a comprehensive measure of overall health status and disease burden (Supplementary Material 1). This index is widely validated and used to predict in-hospital mortality and readmission rates, demonstrating its utility across diverse healthcare settings [27]. In addition, disorders associated with premature birth and low birth weight, as well as pulmonary co-infections were also considered. Specifically, ICD P07x diagnoses and additional classification codes related to bacterial pneumonia (J13-J15), and viral pneumonia (J09, J10.0, and J11.0) were included. Furthermore, the in-hospital mortality was also described. For the analysis of the in-hospital mortality, cases discharged due to hospital transfer or unspecified reasons were excluded.

### Statistical analysis

We extracted administrative data from QlikView (QlikTech, Radnor, Pennsylvania, USA) and performed inferential statistics. Our approach included generalized linear mixed models (GLMM) with hospitals as random factors. The effects were estimated using the lme4 package (version 1.1–26) in the R environment for statistical computing (version 4.0.2, 64-bit build). All mixed models incorporated varying intercepts for the random factor. The significance was determined using two-tailed tests with an α-level of 0.05. Patient characteristics and comorbidities were analyzed using χ^2^-tests for categorical (age group, sex, main diagnosis, SARS-CoV-2 infection and other comorbidities, Elixhauser comorbidity index, and hospital type) and two-sample t-tests for numerical variables (the age and Elixhauer comorbidity score). We reported proportions, means, standard deviations, and *p*-values. In addition, logistic GLMMs were used to compare proportions of treatments and outcomes (intensive care, mechanical ventilation, in-hospital mortality, low/intermediate/high duration of mechanical ventilation). We also computed terciles of duration of mechanical ventilation and used the information in order to create three dichotomous variables (presence of observation in tercile). Proportions, odds ratios (OR), confidence intervals, and *p*-values were reported. For the continuous variable including length of hospitalization and length of stay in ICU, linear mixed models were employed after transforming them via inverse hyperbolic sine to approximate normal distributions (visual inspection). We described means, standard deviations, medians, interquartile ranges, and *p*-values,  and computed *p*-values based on the Satterthwaite Approximation for degrees of freedom. Multivariable analyses of outcomes were performed through logistic GLMM and linear mixed model. Categorical variables entered the multivariable analyses as simple-coding contrasts while the continuous variable age was centered at its mean. All continuous variables were scaled to unit variance. Moreover, we presented statistics for the Elixhauser comorbidity index and its items, including the weighted Elixhauser comorbidity index using the AHRQ algorithm (Supplementary Material 1).

## Results

### Patient characteristics

In this study, 6,816 children with RSV infection were hospitalized between 1^st^ January 2016 and 29th February 2020 (PreCoV19 group), and 5,099 were hospitalized between 1^st^ March 2020 and 31^st^ December 2022 (CoV19 group). In 2020, coinciding with the onset of the COVID-19 pandemic, there was an important decline in the numbers of cases, which was followed by a significant increase in the subsequent year (Fig. 1a).

Among all patients, 6,818 (57.2%) children were male and 5,097 (42.7%) were female, with a similar distribution of sex across both groups. The mean age significantly differed between the PreCoV19 and CoV19 cohorts, where children of the PreCoV19 group were 0.4 ± 0.7 years, and of the CoV19 were 0.6 ± 1.0 years old (*p* < 0.001). A higher age dispersion was observed in children hospitalized during the pandemic. Overall, children under one year of age represented 70.7% (8,429) of all cases. Regarding comorbidities, hospitalized children from the CoV19 group had a significantly higher Elixhauser Comorbidity Index of 2.2 ± 4.4 compared to the PreCoV19 cohort (2.0 ± 4.3; *p* = 0.005) (Supplementary Material 2). Disorders related to short gestation and low birth weight, as defined by a P07x diagnosis, were present in 14 cases (0.2%) in the PreCoV19 cohort and 5 cases (< 0.1%) in the CoV19 cohort (Table 1).

**Table 1:** Summary of patient characteristics

Clinical characteristics	PreCov19 *n* = 6,816	Cov19 *n* = 5,099	*p*-value
Mean age	0.4 (0.7); 0.0 [0.0–1.0]	0.6 (1.0); 0.0 [0.0–1.0]	< 0.001
Age			< 0.001
0 years	5,076 (74%)	3,353 (66%)	
1 year	1,184 (17%)	819 (16%)	
2 years	375 (5.5%)	542 (11%)	
3 years	131 (1.9%)	258 (5.1%)	
4 years	50 (0.7%)	127 (2.5%)	
Sex			0.437
Male	3,879 (57%)	2,939 (58%)	
Female	2,937 (43%)	2,160 (42%)	
Main diagnosis			< 0.001
J12.1	2,525 (37%)	1,463 (29%)	
J20.5	1,766 (26%)	1,306 (26%)	
J21.0	2,525 (37%)	2,330 (46%)	
SARS-CoV-2 infection	0 (0%)	49 (1.0%)	< 0.001
Disorders related to short gestation and low birth weight	14 (0.2%)	5 (< 0.1%)	0.222
Bacterial pneumonia	53 (0.8%)	33 (0.6%)	0.470
Viral pneumonia	16 (0.2%)	14 (0.3%)	0.807
Elixhauser comorbidity index			< 0.001
< 0	50 (0.7%)	18 (0.4%)	
0	5,490 (81%)	4,009 (79%)	
1–4	21 (0.3%)	22 (0.4%)	
≥ 5	1,255 (18%)	1,050 (21%)	
Elixhauser comorbidity score	2.0 (4.3); 0.0 [0.0–0.0]	2.2 (4.4); 0.0 [0.0–0.0]	0.005
Hospital type			< 0.001
Primary care	2,010 (29%)	1,323 (26%)	
Secondary care	2,529 (37%)	2,168 (43%)	
Tertiary care	2,277 (33%)	1,608 (32%)	

Mean (SD); Median [25-75%]; n (%)^*2*^ Welch Two Sample t-test (age, Elixhauser comorbidity score); Pearson’s Chi-squared test (categorical variables)

### Severity of infection

#### Hospitalization time

**Fig. 2:**
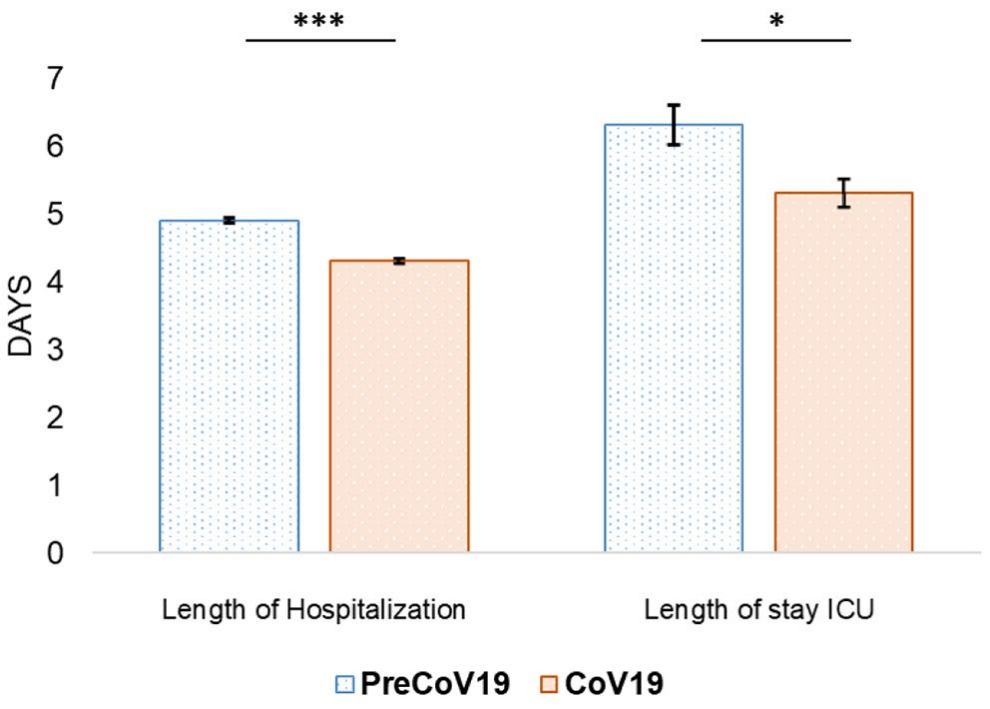
The difference in treatment duration measured as hospitalization time and length of stay at intensive care unit between PreCoV19 and CoV19 cohorts. Bars show mean values. (*p ≤ 0.05, **p ≤ 0.01, ***p ≤ 0.001). The error bars indicate ± standard error

The length of hospital stay revealed significant differences between the groups. In detail, children with RSV infections from the PreCoV19 group had a mean hospitalization duration of 4.9 ± 3.3 days and a median of 4.0 days [3.0; 6.0], with 25% of children being hospitalized for longer than 6 days. In the CoV19 cohort, the mean value of hospitalization time decreased to 4.3 ± 2.6 days, with a median of 4.0 days [2.0; 5.0] (Fig. 2). Moreover, the reduction coefficient of -0.15; 95% CI -0.17; -0.13] in hospitalization time was highly significant (*p* < 0.001). A reduction in the length of stay was observed among children aged 0 to 1 years as well as 2 to 4 years. During the pandemic, the median hospitalization of the latter age group was 3 [3;6] days and a mean value of 4.1 ± 2.9 days compared to 4 [2;5] days, and a mean value of 4.6 ± 3.0 days before COVID-19 (*p* < 0.001). The length of hospital stay was dichotomized into two groups based on the median duration. For longer hospital stays (4 days or more), a significant difference was observed between the pre-pandemic and pandemic periods. In detail, 66% of patients had longer stays during pre-pandemic compared to 53% during pandemic (OR 0.56, 95% CI: 0.52–0.61, *p* < 0.001).

#### Intensity of treatment

**Fig. 3:**
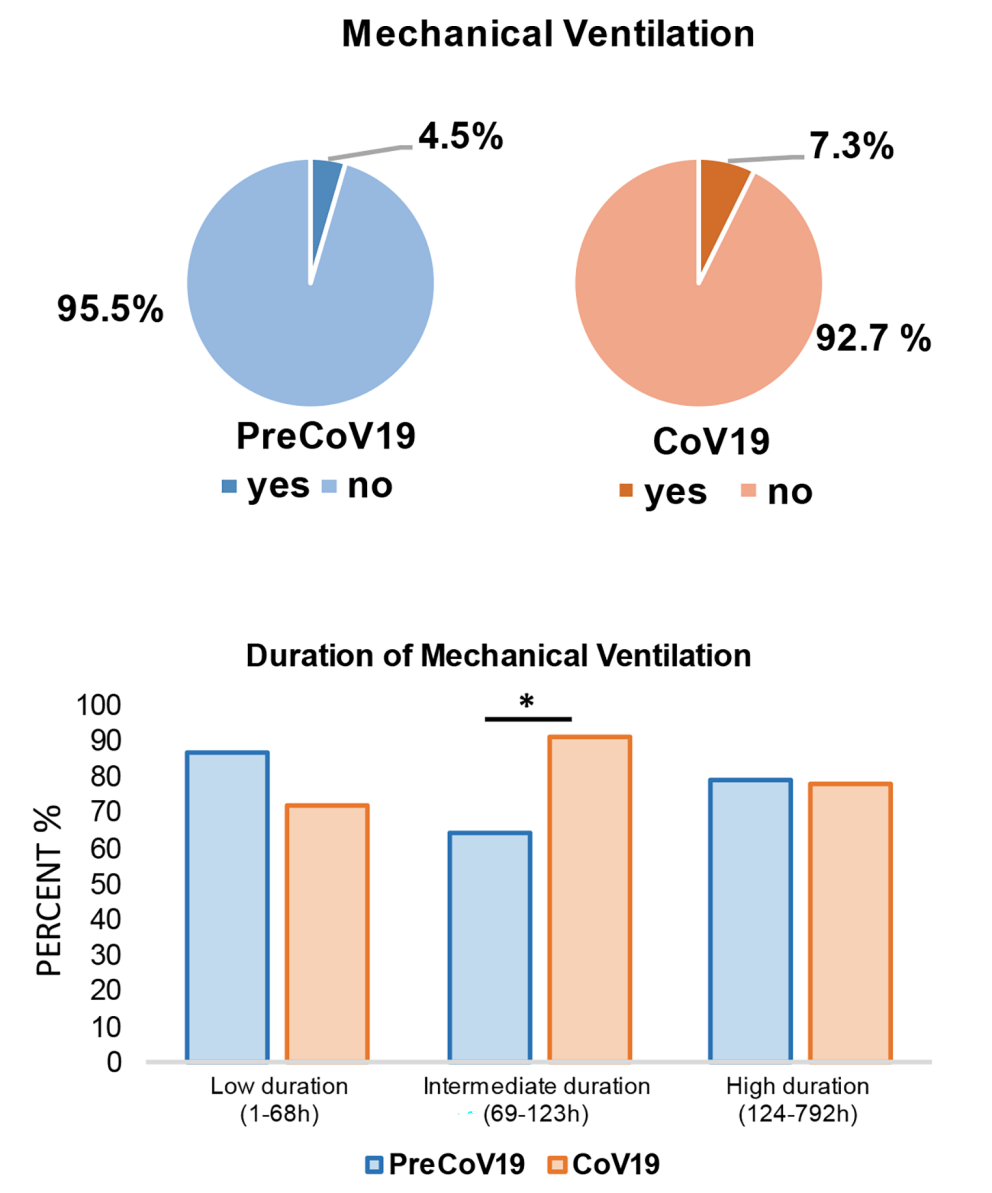
Requirement of mechanical ventilation in children with RSV infection during pandemic. (*p ≤ 0.05)

When comparing both study groups (PreCoV19/CoV19), there was no significant difference in ICU admissions between PreCoV19 (*n* = 389; 5.7%) and CoV19 (*n* = 303; 5.9%) (*p* = 0.84). The majority of ICU admissions in both groups were children who were between 0 and 1 years of age (*n* = 368, 94% of PreCoV19 and *n* = 265; 87% of CoV19). However, significant differences were observed in the length of stay at the ICU. In detail, patients from the PreCoV19 group stayed 6.3 ± 5.8 days in average, whereas children from the CoV19 group were 5.3 ± 3.6 days (*p* = 0.024) at the ICU (Fig. 2). The CoV19 group had a significantly higher likelihood of requiring mechanical ventilation with an OR of 1.5 (CI 1.3; 1.8; *p* < 0.001). Before the COVID-19 pandemic (PreCoV19 group), 4.5% (*n* = 309) of children received mechanical ventilation, compared to 7.3% (*n* = 373) during the pandemic (Fig. 3).

In addition, children aged 0 to 1 years required external oxygen supply with an OR of 1.7 (CI 1.4; 2.0; *p* < 0.001). The comparison of mechanical ventilation duration tertiles (low: 1–68 h, intermediate: 69–123 h, high: 124–792 h) between the PreCoV19 and CoV19 groups revealed less variability in the CoV19 group. An F-test for equality in variances tests confirmed significantly different variances, with the pandemic group showing less variability. This reduced variance might indicate more uniform treatment protocols or a more homogeneous presentation of the disease during the pandemic. Furthermore, there was no significant difference in the low (*p* = 0.564) and high (*p* = 0.318) duration tertiles, but the intermediate duration tertile showed a significant increase during the COVID-19 period (*p* = 0.041) (Table 2).

**Table 2:** Summary of in-hospital outcomes

In-Hospital Outcomes	PreCoV19 *n* = 6,816 (%)	CoV19 *n* = 5,099 (%)	Odds ratio/ coefficient	95% CI	*p*-value
Intensive care	389 (5.7%)	303 (5.9%)	0.98	0.84, 1.2	0.842
Mechanical ventilation (MV)	309 (4.5%)	373 (7.3%)	1.5	1.3, 1.8	< 0.001
In-Hospital mortality	2 (< 0.1%)	2 (< 0.1%)	1.3	0.18, 9.2	0.808
Low duration MV	87 (38%)	72 (30%)	0.88	0.57, 1.4	0.564
Intermediate duration MV	64 (28%)	91 (38%)	1.5	1.0, 2.3	0.041
High duration MV	79 (34%)	78 (32%)	0.81	0.53, 1.2	0.318
Length of stay	4.9 (3.3); 4.0 [3.0–6.0]	4.3 (2.6); 4.0 [2.0–5.0]	-0.15	-0.17, -0.13	< 0.001
Length of stay at ICU	6.3 (5.8); 5.0 [3.0–8.0]	5.3 (3.6); 5.0 [3.0–7.0]	-0.12	-0.23,-0.02	0.024

n (%); Mean (SD); Median [25-75%]^*2*^ CI = Confidence Interval. Dichotomous variables were tested with mixed logistic regression, (transformed) length of hospitalization and length of stay ICU via mixed linear regression

In addition, the selection of diagnosis also varied between the groups. In detail, in the PreCoV19 cohort, 37% (*n* = 2525) of the hospitalized cases received the ICD-code RSV pneumonia (J12.1) compared to 29% (*n* = 1463) in the CoV19 group (*p* < 0.001). Almost half of the cases (*n* = 2330) during the pandemic were diagnosed with RSV-Bronchiolitis (J21.0). However, the in-hospital mortality remained consistent showing an absolute number of two cases in both groups (< 0.1%). In both the PreCoV19 and CoV19 groups, cases of bacterial pneumonia (J13-J15) were diagnosed, of whom in particular, 53 cases (0.8%) were reported from the PreCoV19, and 33 cases (0.6%) from the CoV19 group. Moreover, the incidence of viral pneumonia (i.e., J09, J10.0, J11.0) per co-infection was interestingly less, where 16 cases (0.2%) were in the PreCoV19 and 14 cases (0.3%) in the CoV19 groups. Furthermore, a COVID-19 co-infection has been occurred in 49 cases (1.0%).

#### Multivariable analyses of in-hospital outcomes

**Fig. 4:**
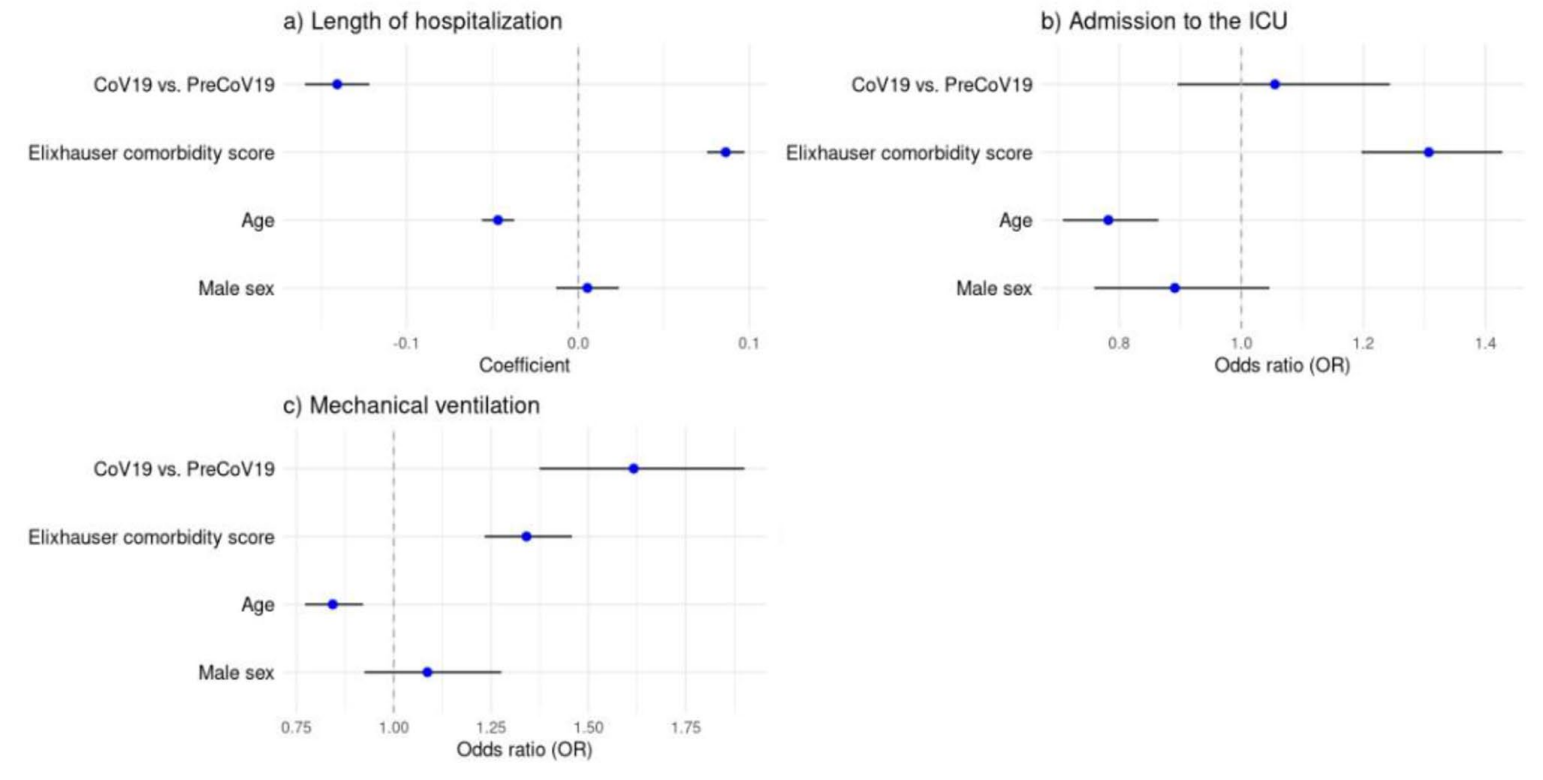
Multivariable analyses of in-hospital outcomes, including coefficients or OR with 95%-Confidence Intervals. Analyzed via linear mixed models and mixed logistic regression

In the multivariable analyses, we then analyzed the role of sex, age, period (pandemic vs. pre-pandemic), and Elixhauser comorbidity scores on in-hospital outcomes. Specifically, a higher age (Coeff. -0.05 [95% confidence interval: -0.06; -0.04]), the correlation with the CoV19 cohort (Coeff. -0.14[-0.16; -0.12]), and a lower comorbidity score (Coeff. 0.09 [0.08; 0.1]) were associated with reduced hospitalization duration (Fig. 4), where these effects were statistically significant (all *p*-values are < 0.001). Moreover, a younger age (OR 0.78 [0.71; 0.86]) and a higher Elixhauser comorbidity score (OR 1.31 [1.20;1.43]) correlated with an increased likelihood of ICU admission (both *p*-values are < 0.001). Interestingly, the age (Coeff. 0.00 [-0.06; 0.06]) was not significantly correlating with the length of stay at ICU, but the hospitalization of PreCoV19 (Coeff. -0.13 [-0.24; -0.03], *p* = 0.015) and a higher Elixhauser comorbidity score (Coeff. 0.07 [0.02; 0.13], *p* = 0.008) correlated with longer stay at ICU. Furthermore, a younger age (OR 0.84 [0.77; 0.92]), a higher Elixhauser comorbidity score (OR 1.34 [1.23; 1.46]), and a hospitalization during the COVID-19 pandemic (OR 1.62 [1.37; 1.90]) were all correlating with higher OR in mechanical ventilation (all *p*-values are < 0.001). In addition, the statistical interaction analyses between age groups and hospitalization periods (pre- or during the pandemic) revealed an effect on the duration of intensive treatment, with a more significant decrease in children aged 2 to 4 years during the pandemic than in those patients aged between 0 and 1 years (Coeff. -0.43 [-0.81; -0.04], *p* = 0.0032). Due to the limited number of cases, there were no analyses of in-hospital mortality performed.

## Discussion

During the COVID-19 pandemic, epidemiological shifts in RSV infections among children led to investigations into transmission dynamics and disease severity [17]. Our study investigates RSV severity in children under five years by analyzing data from a large hospital network between 2016 and 2022. Our findings correlate with observed epidemiological changes, showing a significant increase in RSV incidences during the 2020/2021 and 2021/2022 seasons.

The high burden of RSV incidence rates after the nearly absent circulation in 2020/2021 has been observed in several countries, but studies reported varying findings on patient characteristics and severity [17, 19, 20]. This study adds relevant observations on these aspects by showing shorter hospitalization lengths, constant ICU admission rates, and shorter ICU treatment durations for children with RSV infections during the pandemic. These observations are consistent with a large Canadian study involving 11,014 children analyzing RSV-associated hospitalizations before and during the COVID-19 pandemic [28]. Covering three seasons before and two seasons during the pandemic, there were no significant changes observed in the proportion of children requiring ICU admission or hospitalization for seven days or longer, suggesting that the need for prolonged hospitalization did not increase [28]. Further studies highlighted a decreased requirement for supplementary oxygen, intensive care, or invasive ventilation. A Taiwanese study with 128 pediatric patients during the 2020 to 2021 season demonstrated no significant differences in clinical features including length of hospital stay, oxygen requirements, bronchodilator use, and intensive care treatment [29]. Similarly, Fourgeaud et al. (2021) observed reduced hospitalization times, less frequent ICU admission, a reduced use of non-invasive ventilation and a high-flow nasal oxygen during the 2020/2021 RSV season [30]. In our study, we observed an increased need of mechanical ventilation despite unchanged ICU admission rates. This was possibly due to high-flow oxygen therapy being used outside the ICU. In 2020, the hospital network coding rules changed, allowing high-flow oxygen therapy to be reported in mechanical ventilation hours, which were previously not included.

Among hospitalized patients, few studies showed an increased RSV severity, often requiring an increased oxygen supplementation or ICU admission. In New Zealand, a 2.8 times higher pediatric ICU treatment was reported compared to previous years [31]. A multicenter retrospective study by Movva and colleagues found higher pediatric ICU admission rates (69% vs. 60%) compared to the pre-COVID-19 period [32]. Similarly, a Korean multicenter study among children aged 3 to 24 months reported a significant increase in respiratory support rates in 2021/2022 (19.2%) compared to previous RSV seasons (1.8% in 2017/2018; 6.0% in 2019/2020, *p* = 0.012) [33].

Among the patient characteristics, the CoV19 cohort in our study demonstrated an important shift in age distribution toward older children. Further studies revealed an increased median age of hospitalized children compared to pre-pandemic seasons. Specifically, in the Rhone Loire area, Casalegno et al. (2021) observed an increase in the median age, ranging from 2.2 months before the pandemic to 4.8 months during the delayed RSV season of 2020/2021 [21]. A Danish nationwide cohort study reported no increase in disease severity measured by the need for mechanical ventilation. Furthermore, the authors described increased hospital admission risks for young infants aged ≤ 3 months and children aged 2 to 5 years [22]. These age-specific changes in RSV-associated hospital admissions, compared to previous years, were also supported by additional studies from the U.K., southeast, and Western Australia, reporting increased case numbers for children aged 2 to 4 years [11, 19, 34]. Our results are consisting with these findings, showing increased hospital admission rates among all age groups, with significant peaks in children aged ≤ 1 and those aged ≥ 2 years (Fig. 1). This analysis may introduce the concept of immunity deficit, where children unexposed to RSV during the years 2020 and 2021 showed an increased vulnerability to infections due to their immunological naivety or reduced immunity from the lack of exposure, facilitating rapid transmission [22]. It could be also linked to reduced levels of maternally derived RSV antibodies, as the decrease in RSV exposure among pregnant women was also notable [35, 36].

In addition, the disease severity is associated with age and prematurity [37–39]. Here, a significant proportion of RSV infection morbidity may occur within the first year of life, with among others the INSPIRE study highlighting the burden particularly in preterm infants and those under 6 months, resulting in higher and prolonged ICU admissions [40, 41].

In our study, the older mean age may also contribute to the reduced hospital stay duration, and the consistent rate of pediatric ICU admissions with the shorter treatment duration. The frequency of underlying prematurity nor bacterial or viral co-/superinfections did not significantly differ from the pre-pandemic seasons.

The likely contributing factors and role of the COVID-19 pandemic might be complex. Several factors related to pathogen, host, and environment are not included in this study, which present a limitation. Economic circumstances might have influenced the duration of hospital stay and respiratory treatments due to increased awareness of limited healthcare resources. Thus, shorter hospitalization lengths, stable ICU admission rates, and shorter ICU treatment durations may reflect pandemic-related redistribution of resources, as there are no standardized admission or discharge criteria. Moreover, the increased use of high-flow oxygen therapy might have influenced ICU admission rates, and smaller variation in ventilation hours during the pandemic may suggest a more homogeneous disease presentation or more standardized treatment protocols. Moreover, the pandemic also changed procedures towards viral testing, leading to higher awareness of RSV infections, which could explain epidemiological shifts. Studies from different medical fields report decreased hospital admissions, procedures, operations, shortened hospital stays, fewer cancer diagnoses, and reduced emergency room visits. In addition, the threshold for ICU admissions may have shifted, and the increase in hospitalizations could have influenced the duration of treatments [42–45].

To address this atypical RSV activity, maintaining year-round epidemiological surveillance is thus crucial. Detecting increased cases enable healthcare systems to adapt and plan for potential peaks in hospital burden and disease severity. By understanding these dynamics, we can better prepare for the impact of RSV outbreaks, even beyond the typical seasonal periods. Therefore, expanding these findings to include virus- and environment-related factors can identify criteria for standardized treatment strategies, extending clinical implications beyond pandemic times.

In summary, this study presents a comprehensive dataset from multiple nationwide hospitals across all pediatric care levels. Conducting this multi-center cohort study retrospectively using routine data, the assessment of clinical severity was based on surrogate parameters. This methodology limits deeper analyses and does not allow differentiation between various mechanical ventilation modes, including high-flow oxygen, CPAP, or invasive ventilation. Moreover, the study lacks data on established risk factors for RSV infection severity [46]. Furthermore, the Elixhauser comorbidity index may not fully capture the underlying medical conditions of the included children. Future research should explore viral factors and other clinical determinants, such as low-flow oxygen use, antibiotics, steroids, and laboratory parameters, to provide a more precise assessment of disease severity during pandemic scenarios.

## Conclusion

Our large multicenter cohort analysis of hospitalized children under the age of five with a RSV infection over a 7-year period presents an important additional perspective to existing data on the clinical burden. Epidemiological shifts in RSV seasonality, influenced by non-pharmaceutical interventions during the COVID-19 pandemic, may effect clinical characteristics and management of RSV infections. Our findings demonstrate that RSV-infected children during the pandemic had significantly shorter hospital stays and a reduced ICU treatment duration compared to pre-pandemic children. However, no differences were observed in ICU admission rates or mortality. The probability of requiring mechanical ventilation was higher in hospitalizations during the pandemic. Although well-known risk factors may contribute to disease severity, the specific mechanism behind this still remains unclear. The age of patients, influenced by the COVID-19 pandemic, appears to play a significant role. Further evaluation is needed to understand the broader impact on patient care and viral characteristics. In conclusion, this study highlights the influence of the COVID-19 pandemic on the age distribution, hospitalization rates, and duration of treatment among children infected with RSV.

## Electronic Supplementary Material

Below is the link to the electronic supplementary material.

Supplementary Materials 1 and 2

## Data Availability

No datasets were generated or analysed during the current study.
