# Supplementary material for: The severity of respiratory syncytial virus infection in children during the SARS-CoV-2/COVID-19 pandemic: A nationwide study of 11,915 cases in Germany
Source: Infection. 2024 Sep 10;53(2):561–72. doi: 10.1007/s15010-024-02385-9 (PMC11971059; doi:10.1007/s15010-024-02385-9)
Supplement: Supplementary file 1 — Supplementary Materials 1 and 2 [file 15010_2024_2385_MOESM1_ESM.docx]

**SUPPLEMENTARY MATERIAL**

**Supplementary Material 1. Comorbidities and Algorithm of the Elixhauser Comorbidity Index.**

| **Elixhauser Comorbidity Index** | **AHRQ Algorithm** |
| --- | --- |
| Congestive heart failure | 9 |
| Cardiac arrhythmias | 0 |
| Valvular disease | 0 |
| Pulmonary circulation disorders | 6 |
| Peripheral vascular disorders | 3 |
| Hypertension (combined uncomplicated and complicated) | -1 |
| Paralysis | 5 |
| Other neurological disorders | 5 |
| Chronic pulmonary disease | 3 |
| Diabetes, uncomplicated | 0 |
| Diabetes, complicated | -3 |
| Hypothyroidism | 0 |
| Renal failure | 6 |
| Liver disease | 4 |
| Peptic ulcer disease, excluding bleeding | 0 |
| AIDS/HIV | 0 |
| Lymphoma | 6 |
| Metastatic cancer | 14 |
| Solid tumour without metastasis | 7 |
| Rheumatoid arthritis/collagen vascular diseases | 0 |
| Coagulopathy | 11 |
| Obesity | -5 |
| Weight loss | 9 |
| Fluid and electrolyte disorders | 11 |
| Blood loss anaemia | -3 |
| Deficiency anaemia | -2 |
| Alcohol abuse | -1 |
| Drug abuse | -7 |
| Psychoses | -5 |
| Depression | -5 |

**Supplementary Material 2. Comparison of Comorbidities between the groups.**

| **Comorbidity** | **PreCoV19**  ***n* = 6,816 (%)** | **CoV19**  ***n*= 5,099 (%)** | ***p*-value** |
| --- | --- | --- | --- |
| Congestive heart failure | 12 (0.2%) | 6 (0.1%) | 0.566 |
| Cardiac arrhythmias | 14 (0.2%) | 16 (0.3%) | 0.325 |
| Valvular disease | 8 (0.1%) | 9 (0.2%) | 0.548 |
| Pulmonary circulation disorders | 7 (0.1%) | 2 (<0.1%) | 0.362 |
| Peripheral vascular disorders | 3 (<0.1%) | 6 (0.1%) | 0.267 |
| Hypertension, uncomplicated | 1 (<0.1%) | 0 (0%) | 1.000 |
| Hypertension, complicated | 0 (0%) | 0 (0%) |  |
| Paralysis | 9 (0.1%) | 5 (<0.1%) | 0.791 |
| Other neurological disorders | 88 (1.3%) | 99 (1.9%) | 0.006 |
| Chronic pulmonary disease | 29 (0.4%) | 24 (0.5%) | 0.820 |
| Diabetes, uncomplicated | 0 (0%) | 3 (<0.1%) | 0.156 |
| Diabetes, complicated | 0 (0%) | 0 (0%) |  |
| Hypothyroidism | 15 (0.2%) | 3 (<0.1%) | 0.045 |
| Renal failure | 0 (0%) | 1 (<0.1%) | 0.884 |
| Liver disease | 5 (<0.1%) | 1 (<0.1%) | 0.378 |
| Peptic ulcer disease excluding bleeding | 0 (0%) | 0 (0%) |  |
| AIDS/HIV | 0 (0%) | 0 (0%) |  |
| Lymphoma | 0 (0%) | 0 (0%) |  |
| Metastatic cancer | 0 (0%) | 0 (0%) |  |
| Solid tumour without metastasis | 0 (0%) | 2 (<0.1%) | 0.357 |
| Rheumatoid arthritis/collaged vascular disease | 1 (<0.1%) | 1 (<0.1%) | 1.000 |
| Coagulopathy | 7 (0.1%) | 4 (0.1%) | 0.899 |
| Obesity | 2 (<0.1%) | 2 (<0.1%) | 1.000 |
| Weight loss | 26 (0.4%) | 14 (0.3%) | 0.402 |
| Fluid and electrolyte disorders | 1,147 (17%) | 952 (19%) | 0.010 |
| Blood loss anaemia | 0 (0%) | 0 (0%) |  |
| Deficiency anaemia | 57 (0.8%) | 26 (0.5%) | 0.045 |
| Alcohol abuse | 0 (0%) | 0 (0%) |  |
| Drug abuse | 5 (<0.1%) | 1 (<0.1%) | 0.378 |
| Psychoses | 0 (0%) | 0 (0%) |  |
| Depression | 0 (0%) | 0 (0%) |  |
